# Prospective cohort studies and their contribution to public health end evidence-based medicine

**DOI:** 10.36416/1806-3756/e20250355

**Published:** 2025-09-22

**Authors:** Gustavo Zabert, Cecilia M Patino, Juliana Carvalho Ferreira

**Affiliations:** 1. Facultad de Ciencias Médicas, Universidad Nacional del Comahue, Cipolletti, Argentina.; 2. Methods in Epidemiologic, Clinical, and Operations Research-MECOR-program, American Thoracic Society/Asociación Latinoamericana del Tórax, Montevideo, Uruguay.; 3. Department of Preventive Medicine, Keck School of Medicine, University of Southern California, Los Angeles, CA, USA.; 4. Divisão de Pneumologia, Instituto do Coração, Hospital das Clínicas Faculdade de Medicina, Universidade de São Paulo, São Paulo (SP) Brasil.

## PRACTICAL SCENARIO

In the first half of the XX century, the observation that lung cancer cases appeared to be much more common among smokers led to the suspicion that smoking caused lung cancer. The health consequences of increase prevalence of smoking became a public issue, sparking a scientific controversy over whether the statistical link between smoking and lung cancer was a causal relationship or a mere coincidence.

A cohort study was the decisive factor in resolving the issue.^1^ Some experts argued that a simple correlation did not prove causation, but a carefully designed observational study provided overwhelming evidence that, in this case, it did. The British Doctors Study,^1^ a landmark prospective cohort study that followed over 40,000 male doctors for several decades was crucial for several reasons:

- Temporal sequence: The study established a clear temporal sequence, showing that heavy smoking consistently preceded the diagnosis of lung cancer.
- Strength of association: The study revealed a powerful statistical association. For example, heavy smokers had a risk of developing lung cancer over 20 times that of non-smokers, an effect too large to be easily dismissed as random chance.
- Dose-response relationship: It demonstrated a strong dose-response relationship, proving that the more an individual smoked, the higher the risk became. This systematic increase in risk with increased exposure is a powerful indicator of a causal relationship.

The first scientific publication from the cohort study, published in 1954, was a turning point.^1^ It presented such compelling evidence that it shifted the scientific community from skepticism to a consensus that smoking was a direct and primary cause of lung cancer. 

## WHAT ARE PROSPECTIVE COHORTS?

A cohort study is an observational, longitudinal research design that follows a group of people, or a cohort, over time to see how a specific exposure affects their health outcomes. The core of this design is to compare the risk-the incidence of events-between an exposed group and an unexposed group. Therefore, researchers may calculate the relative risk (RR), which reflects the strength of the association. When the duration of observation is also considered, the incidence rate ratio (IRR) is used to describe the relationship between events and time.^2^

The key feature of this design is that exposure status is established before the disease occurs. A cohort study is called prospective when investigators plan the study and define the variables of interest before enrolling patients and follow them over time.^3^ This method is particularly strong to establish a clear temporal relationship-the exposure is known to have occurred *before* the outcome-and is less prone to bias (Table 1). A retrospective cohort study uses existing records, such as medical records or employment information, to define a cohort and assess past exposure to risk factors. This design is faster and less costly than prospective studies but may suffer from incomplete data and biases such as misclassification of exposure or outcomes.^2^

**Table 1:** Strengths and limitations.

Strengths	Limitations
Temporal Relationship: Establishes the direction of causality (exposure precedes outcome).	Time-Consuming and Expensive: Especially for prospective studies, as they can span many years.
Multiple Outcomes: Can examine the effect of a single exposure on multiple different outcomes (e.g., smoking and lung cancer, heart disease, stroke).	Inefficient for Rare Diseases: Requires a very large cohort and long follow-up period to observe enough cases of a rare disease.
Incidence Rates: Allows for the direct calculation of incidence rates and relative risks.	Potential for Loss to Follow-up: Participants may drop out of the study, which can introduce selection bias if lost individuals differ from those who remain.
Reduces Bias: Less prone to recall bias than case-control studies.	Potential for Confounding Factors: Although confounding variables can be controlled during the analysis, there is always a risk of unmeasured or residual confounding.

The methodology involves:

1. Defining the study population: The cohort should be a representative sample of the population of interest.
2. Defining and measuring the exposure: Researchers accurately define and measure the exposure of interest in all participants at baseline. This may involve surveys, biological markers, or environmental measurements.
3. Follow-up: The cohort is followed over a specified period to monitor the development of the disease. This is typically done through regular check-ups, questionnaires, or linkage to national health databases.
4. Measuring the outcome: The occurrence of the disease or health outcome is systematically and reliably measured in both the exposed and unexposed groups.

## MODERN APPLICATIONS AND FUTURE DIRECTIONS

Cohort studies remain a cornerstone of modern epidemiology. They are used in order to study a wide range of exposures and outcomes, including:

- The long-term effects of environmental pollutants on respiratory health.
- The relationship between dietary patterns and cardiovascular disease.
- The impact of new drug therapies on patient outcomes over time.

Future directions for cohort studies include the integration of advanced technologies, such as genetic and genomic data, to explore the interplay between environmental factors and genetic predispositions. The use of large-scale electronic health records and data linkage will also make it possible to conduct more efficient and comprehensive retrospective cohort studies.

## References

[1] Doll R, Hill AB (2004). The mortality of doctors in relation to their smoking habits a preliminary report. 1954. BMJ.

[2] Grimes DA, Schulz KF (2002). Cohort studies marching towards outcomes. Lancet.

[3] Ferreira JC, Patino CM (2025). Looking back to move forward insights from retrospective cohort studies. J Bras Pneumol.

